# Association Between Depressive Symptoms and Risk of New-Onset Chronic Liver Disease Among Middle-Aged and Elderly Chinese Adults: A Prospective Cohort Study Based on the China Health and Retirement Longitudinal Study

**DOI:** 10.3390/healthcare14131986

**Published:** 2026-07-03

**Authors:** Tiancheng Meng, Yanling Jiang, Guolin Guo, Jianteng Dong, Xiaofan Lai, Jiale Liu, Hongguo Rong, Jian Li

**Affiliations:** 1 School of Traditional Chinese Medicine, Beijing University of Chinese Medicine, Beijing 102000, China; 20250931123@bucm.edu.cn (T.M.); 20230941064@bucm.edu.cn (Y.J.); 20240931103@bucm.edu.cn (G.G.);; 2 Center for Evidence-Based Chinese Medicine, Beijing University of Chinese Medicine, Beijing 102000, China

**Keywords:** depressive symptoms, chronic liver disease, cohort study, CES-D-10, CHARLS

## Abstract

**Background:** Chronic liver disease (CLD) imposes a heavy global burden, particularly among middle-aged and elderly adults. Prospective evidence on whether depressive symptoms independently predict new-onset CLD remains scarce. **Methods:** This prospective cohort study used CHARLS (2011–2020) data and enrolled 9549 participants without baseline CLD. Depressive symptoms were assessed using the CES-D-10 and categorized into four severity levels. Multivariable Cox regression, Kaplan–Meier analysis, and ROC curves were used. Subgroup analyses were conducted across demographic and behavioral strata to assess consistency. **Results:** During a mean follow-up of 8.77 years, 746 (7.8%) participants developed new-onset CLD. Higher baseline depression severity was associated with increased CLD risk. In the fully adjusted model, compared with the non-depressed group, the HR for mild depression was 1.34 (95% CI: 1.12–1.59, *p* = 0.001), for moderate depression 1.65 (95% CI: 1.34–2.04, *p* < 0.001), and for severe depression 2.16 (95% CI: 1.26–3.72, *p* = 0.005). Cumulative incidence increased with depression severity, with group differences widening over time (log-rank *p* < 0.0001). Subgroup analyses showed consistent associations across different demographic and behavioral strata. **Conclusions:** Across the total population, the analyses consistently demonstrate an independent, graded association between baseline depressive symptom severity and incident CLD that persists after multivariable adjustment. The stratified analyses further reveal that this association is primarily driven by female participants and middle-aged adults (45–60 years), highlighting these subgroups as priority populations for depression-informed CLD prevention and screening strategies.

## 1. Introduction

Chronic liver disease encompasses a spectrum of disorders including chronic viral hepatitis, alcoholic liver injury, autoimmune hepatitis, and cryptogenic fibrosis, all of which follow a similar progression trajectory from subclinical inflammation through fibrosis to cirrhosis and its complications [1,2,3,4,5]. The global burden of CLD is substantial: it is estimated that over 1.5 billion people have some form of CLD, with approximately 2 million deaths annually [1,6,7,8]. In China, the high prevalence of hepatitis B intersects with the growing incidence of metabolic and alcoholic liver diseases, creating a unique and complex situation affecting about 300 million people, and the prevalence among middle-aged and elderly populations exceeds 10% [6,9,10,11,12]. A notable feature of early CLD is the absence of obvious clinical symptoms; during the long preclinical phase, a large proportion of cases remain undetected before irreversible liver damage occurs. Given this epidemiological status, developing practical and scalable approaches for early risk identification and population-level prevention strategies has become an urgent public health priority.

A growing body of evidence from cardiovascular, endocrine, and musculoskeletal studies indicates that psychological distress, especially depression, is an independent determinant of chronic disease incidence, operating through interconnected neuroendocrine, immune, and behavioral pathways [13,14,15,16,17]. The natural course of CLD is influenced by the complex interplay of lifestyle, cumulative comorbidity burden, and psychosocial status, which often coexist in individuals and jointly shape long-term health trajectories [15,18,19]. Within this holistic framework, psychological distress, particularly depression, as a modifiable potential factor, has attracted increasing research attention for its association with the development and progression of chronic diseases. Depression is highly prevalent among middle-aged and elderly individuals, with community-based studies in China showing a 20–40% prevalence of depressive symptoms in this age group [14,15,17,20]. Depression has been consistently linked to adverse health outcomes including cardiovascular disease, type 2 diabetes, and all-cause mortality [13,14,15,17,21]. At the biological level, persistent activation of the hypothalamic–pituitary–adrenal axis and sympathetic nervous system in depression leads to chronic hypercortisolemia, systemic low-grade inflammation, and insulin resistance—processes with well-documented hepatotoxic consequences, including hepatic lipid accumulation, oxidative damage, and stellate cell activation [16,22,23]. Behaviorally associated factors of depression, including excessive alcohol consumption, smoking, a sedentary lifestyle, and unhealthy dietary habits, constitute an additional, mechanistically distinct pathway through which depression may promote liver injury [13,15,17,24]. Furthermore, depression may affect healthcare utilization and medication adherence, leading to delayed identification and inadequate treatment of early liver disease [17,19,24,25].

Despite these plausible mechanistic links, prospective evidence linking depressive symptoms to the onset of CLD remains limited in multiple important respects. Several cross-sectional studies have documented a higher burden of depressive symptoms in patients with diagnosed liver disease, but the inherent inability of cross-sectional designs to establish temporal order leaves the direction of the association unclear [26,27,28,29]. In longitudinal studies, Zeng et al. demonstrated that higher depressive symptoms at baseline predicted the development of CLD in CHARLS data up to 2018, but their three-category classification scheme combined asymptomatic presentation and mild symptoms into a single reference category, thus failing to detect increased risk at the mild symptom threshold [26]. A study by Wang et al. supported this finding [30]. Subsequently, researchers modeled CES-D-10 scores as a continuous predictor in a CHARLS-based cohort and confirmed a significant association per score increment (HR: 1.020 per point; 95% CI: 1.006–1.033) [30]. However, this approach assumed uniform incremental risk across the score distribution and did not assess whether clinically recognized severity thresholds could distinguish meaningful risk strata. Neither study evaluated the predictive discriminative ability of depressive status across different time spans, and both were limited by shorter follow-up periods or narrower analytical scopes. In contrast, a four-level categorization (non-depression, mild, moderate, and severe) aligns with established clinical conventions for depression screening, captures potential non-linear or threshold effects across severity strata, enables identification of high-risk subgroups for targeted intervention, reduces sensitivity to minor measurement variability, and enhances comparability with prior epidemiological studies. Therefore, in the present study, depressive symptom severity was categorized into four clinically meaningful levels to provide more informative and translationally relevant risk estimates for incident CLD.

To fill these research gaps, the present study used data from a large, nationally representative cohort of middle-aged and elderly Chinese adults from the CHARLS. Using CHARLS data covering the full observation period from 2011 to 2020, we employed multivariable Cox proportional hazards regression (with depressive symptom severity categorized into four levels), Kaplan–Meier cumulative incidence estimation, and ROC curve analysis at four time points, supplemented by comprehensive subgroup and interaction analyses.

## 2. Materials and Methods

### 2.1. Study Population

**Figure 1:**
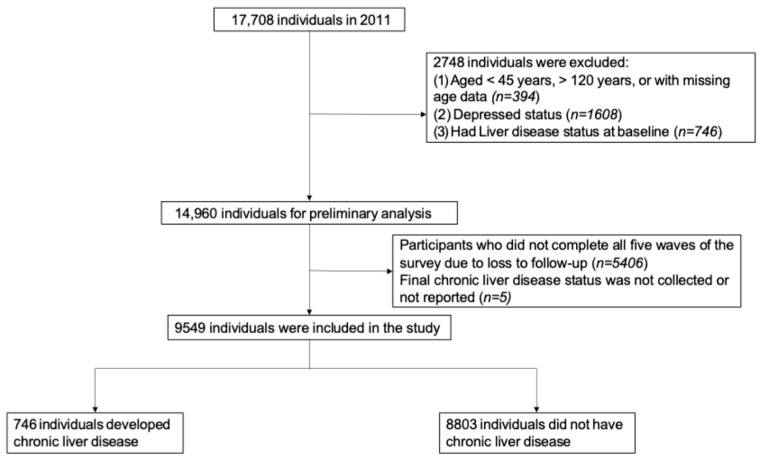
The flowchart of this study.

This prospective cohort study utilized data from the CHARLS (2011–2020), Categorizing CES-D-10 scores into clinically meaningful severity strata (non-, mild, moderate, and severe depression), rather than treating the score as a dichotomous or strictly linear variable, which aligns with how depressive symptoms are recognized and managed in primary care and community screening settings. This stratified approach enables identification of risk gradients at clinically actionable thresholds—particularly the mild symptom level, which is often overlooked yet potentially modifiable—thereby providing more directly translatable evidence for tiered public health screening, targeted intervention allocation, and risk stratification in CLD prevention frameworks. Five waves of follow-up surveys have been completed to date, with participants being followed up every 2–3 years to ascertain their health status. Detailed descriptions of the survey methodology can be found in previous publications [31]. A total of 17,708 individuals were enrolled in the first wave. Participants were excluded if they: (1) were aged <45 years or >120 years, or had missing age information (*n* = 394); (2) had missing baseline depression status data (*n* = 1608); (3) reported having CLD at baseline (*n* = 746). After these exclusions, 2748 participants were excluded, and 14,960 entered the preliminary analysis stage. During follow-up, an additional 5406 participants were excluded due to lack of complete longitudinal data across all follow-up waves, and another 5 were excluded because their final liver disease status could not be determined, leaving 9549 participants for the final analysis. Of these, 746 developed new-onset CLD during follow-up, and 8803 remained free of CLD throughout. Additional information regarding the criteria for participant inclusion and exclusion is detailed in Figure 1. All data are publicly available [31]. The study protocol was approved by the Biomedical Ethics Committee of Peking University (IRB00001052-11015), and all participants provided written informed consent. This report complies with the Strengthening the Reporting of Observational Studies in Epidemiology (STROBE) guidelines for cohort studies.

### 2.2. Assessment of Depressive Symptoms

Depressive symptoms were assessed at baseline in 2011 using the 10-item Center for Epidemiologic Studies Depression Scale (CES-D-10). Each item was rated on a 4-point Likert scale (0–3), and two positively worded items were reverse-coded before summation, yielding a total score ranging from 0 to 30, with higher scores indicating greater symptom burden. Based on previously validated thresholds for Chinese middle-aged and older adults and consistent with classifications widely adopted in CHARLS-based studies, participants were categorized into four severity groups: non-depressed (≤9), mild depression (10–15), moderate depression (16–25), and severe depression (≥26). The cut-off of ≥10 has been validated as the optimal threshold for screening clinically significant depressive symptoms in Chinese community-dwelling middle-aged and elderly populations, demonstrating satisfactory sensitivity and specificity against diagnostic interviews. The further stratification at 15 and 21 follows established conventions used in prior CHARLS-based investigations to differentiate moderate from severe symptom burden, allowing identification of dose–response gradients across clinically actionable severity levels [32].

### 2.3. Ascertainment of Chronic Liver Disease

Incident chronic liver disease (CLD) was ascertained based on participants’ self-reported physician diagnoses collected through the standardized CHARLS health status questionnaire across follow-up waves (2013, 2015, 2018, and 2020). Specifically, the outcome was derived from the item: ‘Have you been diagnosed with liver disease (except fatty liver, tumors, and cancer) by a doctor?’ Participants who answered ‘yes’ at any follow-up wave, having reported no liver disease at baseline (2011), were defined as incident CLD cases. If chronic liver disease had been reported by a participant in any previous wave of the CHARLS survey, the condition was carried forward and the participant was automatically classified as having chronic liver disease in all subsequent waves.

### 2.4. Outcome

During 83,711 person-years of follow-up (mean 8.77 years), the outcome was defined as the time from the baseline interview (2011) to the year of first diagnosis of CLD, the last completed follow-up, or loss to follow-up, whichever came first. Incident CLD was based on the first self-reported physician diagnosis.

### 2.5. Covariates

To preserve the temporal order of exposure and outcome, covariates were ascertained at baseline in 2011. Based on clinical relevance and the published literature, the following variables were included: demographic variables (age and gender), body mass index (BMI); socioeconomic status (residence: rural vs. urban); marital status (married and cohabiting vs. divorced, separated, widowed, or never married); educational level (illiterate, primary education, higher education); health behaviors (smoking: non-smoker vs. smoker; alcohol consumption: non-drinker vs. drinker). Health status was assessed based on limitations in activities of daily living (ADL) and instrumental activities of daily living (IADL). In addition, the number of children was categorized into four levels: childless, one-child, two-child, and multi-child families; mental health status was assessed based on self-reported life satisfaction (satisfied vs. dissatisfied), self-rated health status (healthy vs. unhealthy), and multiple chronic conditions.

### 2.6. Statistical Analysis

For continuous baseline variables, data with a normal distribution are expressed as the mean ± standard deviation (SD) and were compared using the independent-samples *t*-test, while non-normally distributed data are presented as the median (interquartile range, IQR) and were analyzed using the Wilcoxon rank-sum test. Categorical variables are presented as n (%) and were compared using the chi-square test or Fisher’s exact test. All tests were two-sided, with *p* < 0.05 considered statistically significant.

Cox proportional hazards regression models were used. Three models with stepwise adjustment for covariates were fitted: Model 1 (unadjusted); Model 2 (adjusted for age and gender); Model 3 (fully adjusted for all covariates listed in Section 2.5). HRs and 95% CIs for each depressive symptom category were estimated using the non-depressed group as the reference. Tests for linear trend were performed by entering the four-level variable as a single ordinal predictor into the model. The proportional hazards assumption was assessed for all Cox regression models using the Test of Proportional-Hazards Assumption based on Schoenfeld residuals. For all three models, the global test *p*-values were greater than 0.05, and the *p*-values for all individual covariates, including depressive symptom severity, were greater than 0.2, indicating that the proportional hazards assumption was not violated.

Kaplan–Meier survival analysis was used to construct cumulative incidence (failure) curves for each depressive symptom category. The log-rank test was used to compare differences between groups.

To complement the Cox regression findings, ROC analyses were conducted to provide a threshold-based assessment of the discriminative contribution of depressive symptom severity to incident CLD. It should be emphasized that this analysis was not intended to position CES-D-10 as a standalone diagnostic or prognostic instrument for CLD, but rather to evaluate its potential incremental value as a supplementary psychosocial risk marker that could be integrated into existing multifactorial risk stratification frameworks. Given that CLD is influenced by a broad spectrum of metabolic, behavioral, infectious, and genetic determinants, the relatively modest AUC values observed are expected and reflect the inherent limitation of any single exposure to fully discriminate disease risk. Therefore, the ROC results should be interpreted as evidence supporting the complementary—rather than independent—predictive relevance of depressive symptoms for incident CLD.

ROC curve analysis was performed to predict the occurrence of CLD using the four-level depression classification as the predictor at 2-, 4-, 7-, and 9-year follow-up intervals. The area under the curve (AUC) and 95% CI were calculated.

Subgroup and interaction analyses were conducted by fitting fully adjusted Cox models across prespecified strata, including gender, age group, BMI category (<18.5: underweight/18.5–24.9: normal weight/≥25.0: overweight/≥30.0: obesity), smoking status, alcohol consumption, household registration type, educational level, ADL limitation, and IADL limitation. In each stratum, the depression variable was entered as a single ordinal predictor. Effect modification was evaluated by assessing multiplicative interaction terms using the likelihood ratio test.

All analyses were performed using R version 4.2.2 and Stata version 18.0. A two-sided *p* < 0.05 was considered statistically significant.

## 3. Results

### 3.1. Baseline Characteristics of the Cohort

Table 1 presents the baseline characteristics of the 9549 participants, stratified by CLD status during follow-up. The mean age was 58.21 ± 8.67 years, and 54.88% were female. During 83,711 person-years of follow-up (mean 8.77 years), 746 participants (7.8%) developed incident CLD. Notably, participants who developed CLD had a higher prevalence of ADL limitations (20.41% vs. 14.20%; *p* < 0.001) and a higher proportion of multiple chronic conditions (51.74% vs. 35.11%; *p* < 0.001).

### 3.2. Depressive Symptom Severity and New-Onset CLD: Cox Regression Analysis

Table 2 shows the HRs for new-onset CLD across different depressive symptom categories in the total population. In the unadjusted model (Model 1), a clear dose–response relationship was observed between depressive symptom severity and CLD risk. Compared with non-depressed participants, the risk of incident CLD increased progressively across mild (HR = 1.28, 95% CI: 1.08–1.52, *p* = 0.005), moderate (HR = 1.57, 95% CI: 1.29–1.91, *p* < 0.001), and severe (HR = 2.02, 95% CI: 1.20–3.41, *p* = 0.008) depression categories. After adjustment for age and gender (Model 2), the associations remained robust and slightly strengthened (mild: HR = 1.31, 95% CI: 1.10–1.55, *p* = 0.002; moderate: HR = 1.62, 95% CI: 1.33–1.97, *p* < 0.001; severe: HR = 2.16, 95% CI: 1.27–3.65, *p* = 0.004). In the fully adjusted model (Model 3), the dose–response gradient persisted, with mild (HR = 1.23, 95% CI: 1.02–1.48, *p* = 0.030), moderate (HR = 1.40, 95% CI: 1.11–1.77, *p* = 0.004), and severe (HR = 1.85, 95% CI: 1.26–3.72, *p* = 0.033) depression all independently associated with elevated CLD risk, indicating that the relationship is not fully explained by demographic or measured covariates.

### 3.3. Stratified Analysis

Table 2 shows the association between depressive symptom severity and new-onset CLD stratified by gender and age group. In gender-stratified analyses, the positive association was consistent in both genders: in men, mild depression and moderate depression were statistically significant across the three models; in women, mild depression, moderate depression, and severe depression were statistically significant across the three models. In age-stratified analyses, the dose–response gradient was steeper in adults aged 45–60 years, where mild depression, moderate depression, and severe depression were all statistically significant across the three models, while in those aged ≥ 60 years, moderate depression was statistically significant across the three models.

### 3.4. Cumulative Incidence: Kaplan–Meier Analysis

**Figure 2:**
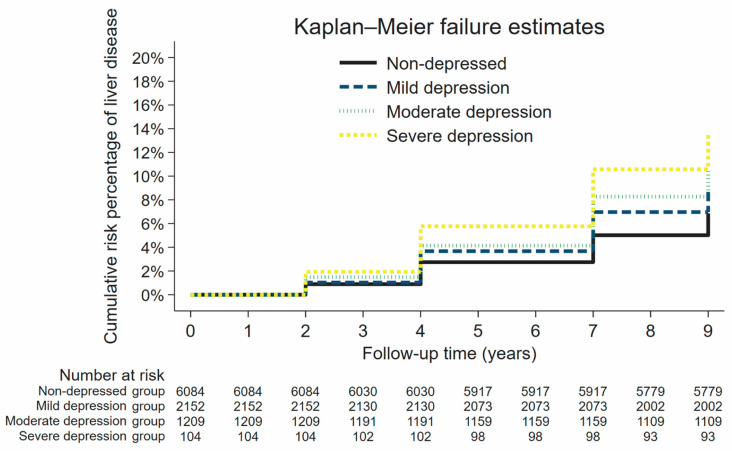
Kaplan–Meier failure curves for cumulative chronic liver disease incidence by baseline depressive symptom category.

Figure 2 shows the cumulative incidence curves of CLD stratified by baseline depressive symptom category. The four curves gradually diverged with longer follow-up, with the lowest cumulative incidence in non-depressed individuals and the highest in severely depressed individuals. The overall log-rank test confirmed significant differences between groups (χ^2^ = 27.99; *p* < 0.0001).

### 3.5. Discriminative Performance: ROC Analysis

**Figure 3:**
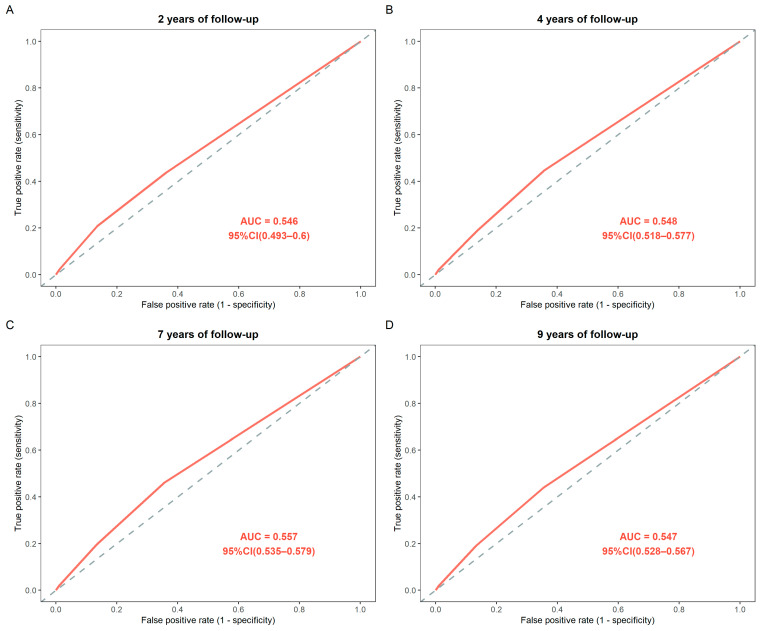
Receiver operating characteristic curves illustrating the predictive performance of baseline depressive status for chronic liver disease risk at different follow-up time points.

Figure 3 presents ROC curves of baseline depressive status as a predictor of CLD at 2-, 4-, 7-, and 9-year follow-up time points. (A) The AUC values were 0.546 (95% CI: 0.493–0.600) at 2 years; (B) 0.548 (0.518–0.577) at 4 years; (C) 0.557 (0.535–0.579) at 7 years, and (D) 0.547 (0.528–0.567) at 9 years.

### 3.6. Subgroup and Interaction Analyses

**Figure 4:**
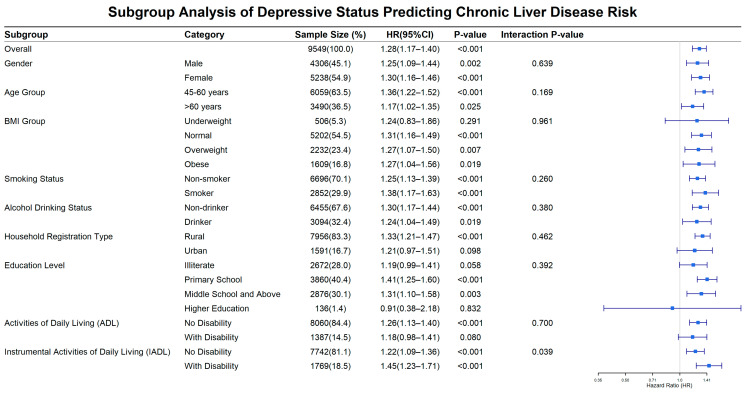
Subgroup analysis of baseline depressive status in predicting chronic liver disease.

In Figure 4, each one-level increase in depression severity is associated with a 28% increased risk of CLD in the total population (HR = 1.28; 95% CI: 1.17–1.40; *p* < 0.001). It was observed that depression severity and CLD risk were not influenced by gender, age, BMI, smoking status, alcohol drinking status, household registration type, education level, or ADL. In other words, the interactions between these variables and depression severity were not statistically significant (*p* > 0.05 for interaction). However, a significant interaction was observed in the IADL subgroup (*p* = 0.039), indicating that IADL status affected the relationship between depression severity and new-onset CLD. Based on the specific estimates, the presence of IADL impairment exerted a certain promotive effect on the association of depression severity with incident CLD.

## 4. Discussion

This prospective cohort study of 9549 middle-aged and elderly Chinese adults yielded three main findings. To begin with, baseline depressive symptom severity (categorized into four levels) was independently and positively associated with the risk of new-onset CLD over a 9-year follow-up period, with graded HRs of 1.34 for mild depression and 2.16 for severe depression in the fully adjusted model. Next, Kaplan–Meier analysis showed that the cumulative incidence of CLD across different depression severity groups gradually diverged over time, with the most pronounced difference emerging after approximately 4 years of follow-up. Furthermore, baseline depressive status served as a predictor, and the consistency of this association across multiple analytical methods and demographic subgroups supported the robustness of these findings. However, given the modest discriminatory performance observed in ROC analysis (AUC range: 0.546–0.557), depressive symptoms should not be regarded as a stand-alone predictor of chronic liver disease. Rather, they highlight the value of incorporating depressive symptom severity as one component within broader, multidimensional CLD risk prediction models in future research.

The present results are consistent with and expand upon the limited but growing longitudinal evidence confirming an association between depressive symptoms and CLD risk [26,30,33]. Previous researchers using CHARLS data up to 2018 found that participants with elevated depressive symptoms (CES-D score ≥ 10) had a significantly higher risk of CLD than those without depressive symptoms, but that study treated depression as a dichotomous exposure and could not analyze the dose–response gradient of symptom severity [26]. Subsequently, another study using CHARLS data modeled CES-D scores as a continuous variable and found that each 1-point increase in depression score was associated with a significantly higher risk of CLD (fully adjusted HR = 1.020), but this study assumed a linear dose–response relationship and did not analyze risk differences across clinically meaningful depression severity levels [26,30].

The present study advances the existing evidence base in several important ways: Firstly, it categorizes depressive symptoms into four ordinal severity levels and demonstrates a stepwise increase in HR with each level (1.34, 1.65, and 2.16 for mild, moderate, and severe depression, respectively), providing direct evidence of a graded correlation. Secondly, our analyses were based on a large nationally representative cohort comprising 9549 middle-aged and older Chinese adults drawn from the China Health and Retirement Longitudinal Study (CHARLS). This substantial sample size provided adequate statistical power to detect clinically meaningful associations between depressive symptom severity and incident CLD. It extends the follow-up period to 2020 (mean 8.77 years, compared with 6.85 years in previous studies), confirming that the association persists over a longer observation window and enhancing confidence in its temporal stability [26,30]. Finally, the present results are broadly consistent with evidence from other populations, with multiple European and international studies also confirming that psychological distress or depression is associated with the incidence of liver-related diseases, particularly NAFLD/MASLD [34,35]. Taken together, these findings suggest that the association between depressive symptoms and CLD may be generalizable across populations, although the strength of the association and underlying mechanisms may vary by regional context [36,37].

The observed association between depressive symptoms and new-onset CLD is biologically plausible and may be mediated by multiple interrelated pathways [38,39].

At the neuroendocrine level, chronic psychological stress activates the hypothalamic–pituitary–adrenal axis and sympathetic nervous system, leading to sustained elevations in circulating cortisol and catecholamines [16,38]. Prolonged cortisol exposure promotes hepatic gluconeogenesis, lipogenesis, and insulin resistance, thereby inducing hepatic steatosis and fibrosis [38,39,40]; meanwhile, excessive sympathetic activation may directly promote hepatic stellate cell activation, a key step in the hepatic fibrosis cascade [40].

At the immune level, depression is associated with chronic low-grade systemic inflammation, characterized by elevated levels of inflammatory markers such as interleukin-6, tumor necrosis factor-α, and C-reactive protein [41,42,43,44]. These inflammatory mediators cause hepatocellular injury, exacerbate oxidative stress in the hepatic microenvironment, and accelerate progression from subclinical hepatic inflammation to clinically overt liver disease [45].

At the behavioral level, individuals with depressive symptoms are more likely to exhibit excessive alcohol consumption, smoking, physical inactivity, and poor dietary quality—all established risk factors for CLD [24]. Although the present models adjusted for baseline smoking and alcohol status, residual confounding from unmeasured dimensions of these behaviors or other lifestyle factors cannot be ruled out [24,35].

Furthermore, emerging evidence based on the gut–liver–brain axis suggests that depression-related alterations in the gut microbiota and impaired intestinal barrier integrity may increase the translocation of bacterial endotoxins into the portal circulation, triggering inflammatory responses in the liver [46,47,48,49,50,51,52]. Although this mechanism remains speculative in the present observational study, it provides a promising direction for future mechanistic research.

It should be emphasized that this study was not designed to disentangle causal mechanisms. The correlation captured between depressive symptoms and CLD likely reflects combined contributions from multiple overlapping pathways rather than a single dominant biological route.

The graded independent association between depressive symptom severity and CLD risk highlights the potential value of integrating routine mental health screening into comprehensive liver disease prevention strategies. These observations further advance the paradigm shift in chronic disease management toward acknowledging bidirectional interconnections between psychological and physical well-being. Incorporating mental health evaluation into routine CLD risk stratification aligns with the growing emphasis on holistic, person-centered interventions for aging populations living with chronic conditions.

The present study has several methodological strengths that advance the existing evidence beyond previous work. First, the nationally representative sampling design of CHARLS, combined with an 80.5% baseline response rate, provides a solid foundation for population-level inference. Second, the mean follow-up time of 8.77 years across four assessment waves represents the longest observation window used to date for this research question within the CHARLS framework, enhancing statistical power for event detection and allowing evaluation of discriminative performance over a longer time frame. Third, the four-level depression classification is more refined than the binary or three-level schemes used in previous studies, enabling detection of increased risk at the mild symptom threshold. Fourth, the multi-method analytical framework—integrating Cox regression, Kaplan–Meier estimation, ROC analysis, and comprehensive subgroup assessment—provides convergent evidence from multiple complementary perspectives, ensuring a comprehensive and internally consistent evaluation of the studied association with both clinical interpretability and statistical flexibility. Fifth, the prespecified and systematic testing of nine interaction terms enhances the credibility of subgroup results and reduces the risk of selective reporting.

Several limitations warrant honest acknowledgment. First, CLD was ascertained via self-reported physician diagnosis rather than objective biochemical markers (e.g., alanine transaminase, aspartate transaminase, γ-glutamyl transferase) or imaging examinations. This approach is prone to misclassification: individuals with more frequent medical visits may be more likely to receive and report a diagnosis, leading to detection bias. Second, detection bias is plausible, as individuals with depression may either have more frequent healthcare contact—facilitating incidental liver disease diagnosis—or, conversely, delay care due to reduced motivation or social withdrawal, leading to underdiagnosis; adjustment for healthcare utilization indicators only partially mitigates this concern. Third, because baseline exclusion relied on self-reported physician diagnosis, participants with subclinical or undiagnosed liver disease (e.g., early NAFLD, asymptomatic chronic hepatitis B/C, early fibrosis) may have been inadvertently included, raising the possibility of reverse causation given the shared risk factors and pathophysiological pathways (e.g., systemic inflammation, gut–liver–brain axis) between depression and liver disease; sensitivity analyses excluding events within the first two years yielded consistent results, but this concern is not fully eliminated. Fourth, the cohort consisted primarily of Chinese individuals, which may limit the generalizability of the findings to other transnational and ethnic groups. Taken together, these limitations suggest that our findings should be interpreted as evidence of a robust association rather than a confirmed causal relationship, and future studies employing objective liver disease assessments, structured psychiatric diagnoses, and causal inference approaches (e.g., Mendelian randomization, target trial emulation) are warranted to further clarify this relationship.

## 5. Conclusions

In this study of 9549 Chinese adults aged 45 years and older, after a mean follow-up of 8.77 years, baseline depressive symptom severity was independently and dose-responsively associated with an increased risk of new-onset CLD. This association remained robust after comprehensive covariate adjustment and was consistent across all demographic and behavioral subgroups. However, given the modest discriminative performance observed in the ROC analyses, depressive symptoms should not be regarded as a stand-alone predictor of CLD. Rather, our findings suggest that assessment of depressive symptoms could be considered as one component of a broader, multidimensional risk assessment framework—alongside established demographic, metabolic, behavioral, and clinical risk factors—to support more comprehensive identification of older adults at elevated risk. Further prospective studies are warranted to evaluate whether incorporating depressive symptom screening into existing risk-stratification tools yields meaningful improvements in predictive accuracy and clinical utility. The studies should verify these findings using objective measurements, explore potential mediating mechanisms, and evaluate the incremental value of depression screening in multivariate CLD risk stratification models.

## Figures and Tables

**Table 1:** Baseline characteristics of the study population.

Characteristics	Total	No Chronic Liver Disease	With Chronic Liver Disease	*p* Value
	*n* = 9549	*n* = 8803	*n* = 746	
Continuous Variables (Mean ± SD)				
Age (years)	58.00 ± 13.00	57.00 ± 13.00	58.00 ± 12.00	0.183
BMI (kg/m^2^)	23.19 ± 4.94	23.16 ± 4.89	23.55 ± 4.93	0.003
Categorical Variables [n (%)]				
Depressive Status				<0.001
Non-depressed	6084 (63.67%)	5669 (64.38%)	415 (55.90%)	
Mild depression	2152 (22.54%)	1965 (22.32%)	187 (25.07%)	
Moderate depression	1209 (12.66%)	1080 (12.28%)	129 (17.16%)	
Severe depression	104 (1.09%)	90 (1.02%)	14 (1.88%)	
Age Group				0.125
45–60 years	6059 (63.45%)	5595 (63.67%)	452 (60.86%)	
>60 years	3490 (36.55%)	3208 (36.33%)	292 (39.14%)	
Gender				0.086
Male	4306 (45.12%)	3950 (44.86%)	362 (48.12%)	
Female	5238 (54.88%)	4853 (55.14%)	384 (51.88%)	
Household Registration Type				<0.001
Rural	7956 (83.34%)	7383 (83.76%)	576 (78.28%)	
Urban	1591 (16.66%)	1420 (16.24%)	163 (21.72%)	
Educational Level				0.002
Illiterate	2672 (28.00%)	2490 (28.48%)	165 (22.28%)	
Primary school	3860 (40.44%)	3541 (40.22%)	319 (43.09%)	
Secondary education	2876 (30.13%)	2635 (29.92%)	241 (32.62%)	
Tertiary education	136 (1.42%)	122 (1.38%)	15 (2.01%)	
Smoking Status				0.627
Non-smoker	6696 (70.13%)	6171 (70.06%)	528 (70.91%)	
Smoker	2852 (29.87%)	2632 (29.94%)	217 (29.09%)	
Alcohol Consumption Status				0.530
Non-drinker	6455 (67.60%)	5943 (67.51%)	507 (68.63%)	
Drinker	3094 (32.40%)	2860 (32.49%)	227 (31.37%)	
ADL				<0.001
No impairment	8060 (85.32%)	7553 (85.80%)	585 (79.59%)	
Impairment	1387 (14.68%)	1250 (14.20%)	153 (20.41%)	
IADL				0.068
No impairment	7742 (81.40%)	7184 (81.61%)	585 (78.90%)	
Impairment	1769 (18.60%)	1619 (18.39%)	157 (21.10%)	
Marital Status				0.358
Currently married	8200 (85.87%)	7541 (85.78%)	652 (87.00%)	
Others	1349 (14.13%)	1262 (14.22%)	96 (13.00%)	
Self-reported Health				<0.001
Good	7415 (77.69%)	6793 (77.18%)	645 (83.78%)	
Poor	2129 (22.31%)	2010 (22.82%)	96 (16.22%)	
Life Satisfaction				0.007
Low	7376 (84.47%)	7464 (84.78%)	599 (80.90%)	
High	1356 (15.53%)	1339 (15.22%)	142 (19.10%)	
Chronic Disease Category				<0.001
No chronic disease	3293 (34.49%)	3040 (34.51%)	176 (23.73%)	
Single chronic disease	2879 (30.15%)	2674 (30.38%)	183 (24.53%)	
Multiple chronic diseases	3377 (35.36%)	3089 (35.11%)	387 (51.74%)	
Number of Children Category				0.057
No child	2495 (26.13%)	2302 (26.14%)	186 (24.66%)	
One child	2291 (24.09%)	2141 (24.31%)	170 (22.39%)	
Two children	2073 (21.71%)	1913 (21.73%)	195 (26.01%)	
Multiple children	2690 (28.17%)	2447 (27.82%)	202 (26.94%)	
Exercise Intensity Group				0.020
No exercise	873 (9.14%)	804 (9.13%)	70 (9.29%)	
Light exercise	2069 (21.67%)	1952 (22.07%)	146 (19.50%)	
Moderate exercise	2878 (30.14%)	2735 (31.07%)	189 (25.08%)	
Vigorous exercise	3729 (39.05%)	3312 (37.73%)	347 (46.13%)	

Data are presented as median (IQR) or n (%). CLD: chronic liver disease; BMI: body mass index; ADL: activities of daily living.

**Table 2:** Cox regression models for the association between the depressive symptom categories and chronic liver disease.

Groups	Depressive Status	Model 1 (Unadjusted)		Model 2 (Adjusted for Age/Gender)		Model 3 (Fully Adjusted)	
		HR (95% CI)	*p* Value	HR (95% CI)	*p* Value	HR (95% CI)	*p* Value
Total Population	Non-depression (Ref.)	1.00 (Reference)	-	1.00 (Reference)	-	1.00 (Reference)	-
	Mild depression	1.28 (1.08–1.52)	0.005	1.31 (1.10–1.55)	0.002	1.23 (1.02–1.48)	0.030
	Moderate depression	1.57 (1.29–1.91)	<0.001	1.62 (1.33–1.97)	<0.001	1.40 (1.11–1.77)	0.004
	Severe depression	2.02 (1.20–3.41)	0.008	2.16 (1.27–3.65)	0.004	1.85 (1.05–3.25)	0.033
Male	Non-depression (Ref.)	1.00 (Reference)	-	1.00 (Reference)	-	1.00 (Reference)	-
	Mild depression	1.29 (1.00–1.66)	0.048	1.29 (1.00–1.66)	0.049	1.18 (0.90–1.56)	0.237
	Moderate depression	1.54 (1.12–2.11)	0.008	1.54 (1.12–2.12)	0.008	1.41 (0.97–2.04)	0.069
	Severe depression	1.85 (0.46–7.51)	0.389	1.85 (0.46–7.52)	0.389	2.19 (0.52–9.31)	0.287
Female	Non-depression (Ref.)	1.00 (Reference)	-	1.00 (Reference)	-	1.00 (Reference)	-
	Mild depression	1.34 (1.06–1.69)	0.016	1.32 (1.05–1.68)	0.020	1.28 (0.99–1.65)	0.057
	Moderate depression	1.69 (1.31–2.19)	<0.001	1.67 (1.29–2.16)	<0.001	1.43 (1.06–1.94)	0.020
	Severe depression	2.27 (1.28–4.02)	0.005	2.23 (1.25–3.97)	0.006	1.77 (0.95–3.30)	0.071
45–60 Years	Non-depression (Ref.)	1.00 (Reference)	-	1.00 (Reference)	-	1.00 (Reference)	-
	Mild depression	1.41 (1.14–1.75)	0.002	1.46 (1.17–1.81)	0.001	1.30 (1.03–1.65)	0.027
	Moderate depression	1.63 (1.26–2.12)	<0.001	1.73 (1.33–2.24)	<0.001	1.39 (1.02–1.89)	0.039
	Severe depression	2.72 (1.47–5.03)	0.002	3.01 (1.61–5.61)	0.001	2.26 (1.15–4.44)	0.018
>60 Years	Non-depression (Ref.)	1.00 (Reference)	-	1.00 (Reference)	-	1.00 (Reference)	-
	Mild depression	1.08 (0.82–1.43)	0.573	1.09 (0.83–1.44)	0.538	1.13 (0.83–1.52)	0.438
	Moderate depression	1.44 (1.07–1.94)	0.015	1.46 (1.08–1.97)	0.013	1.42 (1.00–2.03)	0.050
	Severe depression	1.19 (0.45–3.17)	0.730	1.22 (0.45–3.26)	0.697	1.45 (0.52–4.10)	0.479

Model 1: unadjusted. Model 2: adjusted for age and gender. Model 3: adjusted for Model 2 plus BMI, smoking status, alcohol consumption, household registration type, educational level, marital status, number of children, life satisfaction, self-rated health status, multiple chronic conditions, activities of daily living, and instrumental activities of daily living. HR: hazard ratio; CI: confidence interval.

## Data Availability

The CHARLS data used in this study are available from the official website of the survey (https://charls.charlsdata.com/ accessed on 18 September 2024).
